# An unusual case of OD-allotwinning: 9,9′-(2,5-dibromo-1,4-phenylene)bis[9*H*-carbazole]

**DOI:** 10.1107/S2052520616018291

**Published:** 2017-01-31

**Authors:** Paul Kautny, Thomas Schwartz, Berthold Stöger, Johannes Fröhlich

**Affiliations:** a Institute of Applied Synthetic Chemistry, TU Wien, Vienna, Austria; b Institute of Chemical Technologies and Analytics, TU Wien, Vienna, Austria

**Keywords:** twinning, allotwinning, order–disorder polytypism

## Abstract

9,9′-(2,5-Dibromo-1,4-phenylene)bis[9*H*-carbazole] (1) crystallizes as a category I order–disorder (OD) structure composed of non-polar layers of one kind with *B*2/*m*(1)1 layer symmetry. The crystals are made up of the two polytypes with a maximum degree of order (MDO). The monoclinic MDO_1_ polytype (*B*2_1_/*d*) possesses an orthorhombic *B*-centered lattice and appears in two orientations, which are related by reflection at (100). The orthorhombic MDO_2_ polytype (*F*2*dd*) has a doubled *b*-axis and appears in two orientations, which are related by inversion. The crystal structures of both polytypes were determined in a concurrent refinement. The MDO_1_:MDO_2_ ratio is 69:31.

## Abstract 2

The title compound crystallizes as allotwins made up of two OD-polytypes with a maximum degree of order.

## 1. Introduction   

A twin is a heterogeneous edifice made up of homogeneous crystals of the same phase in different orientations related by an operation that does not belong to the point group of the individual (Friedel, 1904 ▸). The crystalline domains diffract independently and the orientation relationship is well defined (Hahn & Klapper, 2006 ▸). Allotwinning (Nespolo *et al.*, 1999 ▸) is a generalization of twinning, where the domains are different polytypes of the same compound (Greek 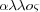 = different) composed of equivalent layers. In contrast to the structural characterization of classical twins, the concurrent refinement of two or more structural models against the same data set is not yet routine. It has nevertheless been performed successfully in a few cases (*e.g.* Friese *et al.*, 2003 ▸; Krüger *et al.*, 2009 ▸; Jahangiri *et al.*, 2013 ▸; Stöger *et al.*, 2015 ▸).

The importance of applying such refinement strategies was shown in recent work by our group on the allotwinning of potassium silver carbonate (KAgCO_3_; Hans *et al.*, 2015 ▸). Therein, we argued that not only the frequency of allotwinning, but also the volume fraction of the minor domain in allotwins tend to be significantly underestimated.

**Figure 1:**
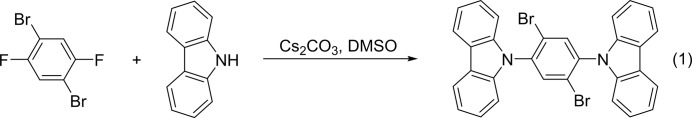
Synthesis of 9,9′-(2,5-dibromo-1,4-phenylene)bis[9*H*-carbazole] (1).

In a continuation of this work, we present an unusual case of allotwinning, which we serendipitously discovered during routine structural analysis of organic intermediates. 9,9′-(2,5-Dibromo-1,4-phenylene)bis[9*H*-carbazole] (1) was synthesized as an intermediate for further functionalization towards functional organic materials for potential applications in organic electronics (Fig. 1 ▸). The two bromine substituents allow for chemical modification *via* Pd catalyzed cross-coupling reactions. Crystals of (1) turned out to be allotwins made up of two members of an order–disorder (OD) polytype family  (Dornberger-Schiff & Grell-Niemann, 1961 ▸). They can, therefore, be designated as *OD-allotwins* in analogy to *OD-twins*, which are made up of different orientations of the same OD-polytype. A non-OD allotwin would be composed of polytypes that do not follow the principles of OD theory.

To encourage future structural characterization of allotwins, a detailed account of data treatment and refinement will be given. Furthermore, an interpretation according to the OD theory is presented, to plausibly explain the polytypism of the compound. Finally, the crystals of (1) are compared with those of KAgCO_3_, to highlight the diversity of the phenomenon.

## 2. Experimental   

### 2.1. Synthesis and crystal growth   

Compound (1) was synthesized by twofold nucleophilic aromatic substitution starting from 1,4-dibromo-2,5-difluoro­benzene and 9*H*-carbazole. A round-bottom flask was charged with 1,4-dibromo-2,5-difluorobenzene (1.09 g, 4.00 mmol, 1.0 eq.), 9*H*-carbazole (1.34 g, 8.00 mmol, 2.0 eq.), Cs_2_CO_3_ (2.87 g, 8.80 mmol, 2.2 eq.) and DMSO (25 ml). The reaction mixture was heated to 393 K for 48 h. After cooling to room temperature the reaction mixture was poured on water (450 ml) and the resulting suspension was filtered. The residue was washed with water and dried under reduced pressure. Purification was accomplished by crystallization from toluene yielding (1) (0.90 g, 1.59 mmol, 40%) as tiny plates, which were used for single-crystal diffraction.

^1^H NMR (400 MHz, CD_2_Cl_2_): δ = 8.20 (d, *J* = 7.7 Hz, 4H), 8.05 (s, 2H), 7.51 (dd, *J* = 8.2, 7.0 Hz, 4H), 7.37 (dd, *J* = 7.6, 7.4 Hz, 4H), 7.28 (d, *J* = 8.2 Hz, 4H) p.p.m. ^13^C NMR (100 MHz, CD_2_Cl_2_): δ = 141.1, 138.8, 136.6, 126.8, 124.1, 123.8, 121.2, 121.1, 110.6 p.p.m. NMR spectra were recorded on a Bruker Avance DRX-400 spectrometer.

### 2.2. Data collection   

Crystals were selected under a polarizing microscope, embedded in perfluorinated oil and attached to Kapton® micromounts. They were subjected to short scans at 100 K in a dry stream of nitrogen on a Bruker KAPPA APEX II CCD diffractometer system (Bruker, 2014 ▸) using graphite-monochromated Mo *K*α radiation. All crystals featured weak diffraction, with a sharp intensity drop off at higher diffraction angles and distinct streaking. A full data set up to 2θ = 55° of the crystal giving the best diffraction pattern was collected with long exposure. A 0.7 mm collimator ensured a full immersion of the platy (0.36 × 0.24 × 0.02 mm) crystal in the X-ray beam. Data were integrated using *SAINT-Plus* (Bruker, 2014 ▸). An adequate correction for absorption effects was performed by using the multi-scan approach implemented in *SADABS* (Bruker, 2014 ▸).

After a first successful refinement, data of four more crystals were collected. Unfortunately, all of them featured even poorer reflection quality and were unfit for a proper structural characterization.

### 2.3. Structure solution and refinement   

**Figure 2:**
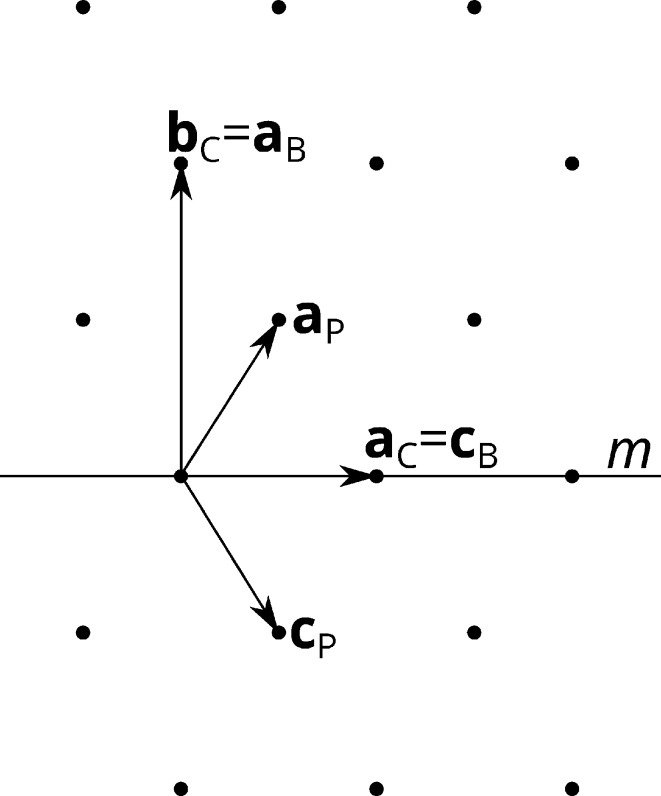
The lattice of the crystal under investigation projected along the monoclinic axis of the major polytype and the bases used during refinement. The horizontal line is the twin plane.

The automatic unit-cell determination suggested an orthorhombic base-centered (*oS*) Bravais lattice (Burzlaff & Zimmermann, 2006 ▸). In the conventional *C*-centered setting 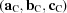 it had the cell parameters *a* ≃ 9.1 Å, *b* ≃ 14.5 Å, *c* ≃ 16.9 Å. The rather large *R*_int_ = 0.082 value for the *mmm* Laue class was attributed to the poor reflection quality. No structure solution was successful even in the lowest symmetry orthorhombic space groups. Likewise, attempts using any space group belonging to the monoclinic *C*-centered (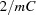) Bravais flock (*R*_int_ = 0.068) were unsuccessful. Thus, the lattice basis was transformed into the primitive setting 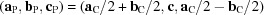, *a* ≃ 8.58 Å, *b* ≃ 16.9 Å, *c* ≃ 8.58 Å, β ≃ 116° (Fig. 2 ▸). Assuming a space group of the monoclinic primitive (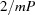) Bravais flock, systematic absences suggested the space group 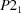. Owing to streaking in the 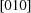 direction, relying on absences is treacherous, because the streaks are interpreted by the integration software as positive Bragg intensity. A structure solution in 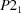 with the novel dual space method implemented in *SHELXT* (Sheldrick, 2015 ▸) did not converge to a usable result, as is common for the intensity data of twins. The direct methods implemented in *SHELXS* (Bruker, 2014 ▸), on the other hand, successfully located the Br atoms and most of the remainder of the molecule. Having succeeded in obtaining a preliminary structural model, no further attempts in triclinic space groups were undertaken.

Being aware of the orthorhombic metrics, the first refinements were performed under consideration of twinning by metric merohedry. The twin plane is indicated in Fig. 2 ▸. In the 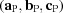 *oP* setting it is parallel to 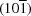; in the 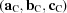 *oC* setting parallel to (100). H atoms were placed at calculated positions and henceforth refined as riding on the parent C atoms. Visual inspection of the structure suggested the higher 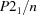 symmetry [the (1) molecule being located on a center of inversion], which was confirmed by the ADDSYM routine of *PLATON* (Spek, 2009 ▸; 100% fit). The correctness of the higher symmetry, which was used for subsequent refinements, was proven by reasonable anisotropic atomic displacement parameters (ADPs) of all non-H atoms, in contrast to the 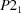 model.

Nevertheless, the residuals were disappointing (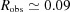) and a prominent peak in the difference Fourier map (ρ = 7.68 e Å^−3^, charge = 2.36 e) was observed close to the carbazole moiety. The coordinates of this peak were approximately 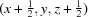 with respect to the coordinates 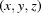 of the Br atom. Since Br is the only heavy atom in the structure, this suggested a spurious ‘phantom’ or ‘shadow’ molecule, which is characteristically observed for structures with improperly resolved stacking faults. The Br atom was refined as positionally disordered between the original position and the ‘phantom’ (refined ratio ∼ 92:8), resulting in a significant drop of the residuals (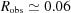).

In many, if not most, cases polytypism is plausibly explained by pseudo-symmetry of distinct layers, called order–disorder (OD) layers  (Grell, 1984 ▸). Indeed, the structure is built of such layers, which are pseudo-symmetric by reflection at 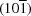 with respect to the *oP* setting (the deviation from perfect symmetry is quantified in §3.4). The linear part of this reflection relates the orientation of the twin individuals. Application of this operation to an adjacent layer maps the positions of the Br and the phantom-Br in that layer.

The Bravais lattice of the OD layers was rectangular base-centered (*os*, note the lower case Bravais letter), with the non-primitive basis 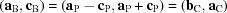 (Fig. 2 ▸). To simplify the OD description, the basis was therefore transformed into the corresponding *B*-centered setting 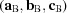. In this setting the symbol of the space group is 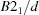, because the intrinsic translation vector of the glide reflection is a quarter of the 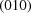 face-diagonal of the unit cell. The twin plane is parallel to 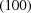. It has to be noted that the Hermann–Mauguin symbol 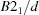 is ambiguous with respect to the orientation of intrinsic translations. Here it designates the group with the *d* glide reflection with intrinsic translation vector 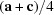, but not 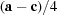.

To explain the ‘shadow atoms’, an interpretation according to OD theory (Dornberger-Schiff & Grell-Niemann, 1961 ▸) was performed (for details, see §3.2). The most probable minor polytype was determined to possess the orthorhombic *F*2*dd* symmetry with a doubled *y*-axis, *i.e.* the lattice basis 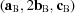. The corresponding reflections, albeit weak and diffuse, indeed exist and were missed by the automatic unit cell determination. Thus, data reduction and absorption correction were repeated with respect to the *B*-centered cell with the basis 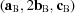. The corresponding reciprocal basis defines the smallest unit cell in which the reflections of both polytypes can be indexed with integral *hkl* values (§3.5).

The occupational disorder of the Br atom was removed from the model of the 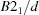 polytype. A model of the *F*2*dd* polytype was generated from the model of the 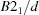 polytype by moving the origin to 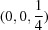, halving the *y*-coordinates and applying the *F*2*dd* symmetry. This model featured a chemically implausibly bent dibromobenzene fragment. To achieve a reasonable model, the atoms of this fragment were all placed onto the 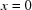 *d* glide plane (but not fixed there in subsequent refinements).

The models of both polytypes were then combined and refined against the same data set using the *JANA*2006 (Petříček *et al.*, 2014 ▸) software. As previously, the 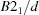 model was refined with two orientations related by reflection at (100). The *F*2*dd* model was refined as two orientations related by inversion. Thus, in total the intensity data was calculated as originating from four independently diffracting domains. The C atoms in the minor *F*2*dd* polytype were refined with isotropic ADPs. The refinements converged quickly to satisfying residuals (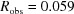).

In diffraction patterns of crystals with a high stacking fault probability, a systematic misevaluation of intensities owing to different shape and backgrounds of different reflection classes may occur. It can lead to erroneous volume fractions of the polytypes (Ďurovič effect; Nespolo & Ferraris, 2001 ▸). In the crystal under investigation, reflections *hkl* with 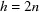, 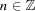 (or equivalently 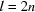) are distinctly elongated and located on rods of diffuse scattering (§3.5). To at least partially counteract this effect, for reflections 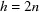 and 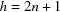 different scale factors were used. Although the refined volume ratios changed only within the estimated standard uncertainty (e.s.u.) range, the residuals improved distinctly (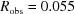). Moreover, the highest peaks in the difference Fourier maps of both polytypes were less pronounced (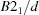: 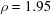 *versus* 1.47 e Å^−3^, charge = 1.40 *versus*  0.90 e; *F*2*dd*: 2.16 *versus*  1.76 e Å^−3^, charge = 0.05 *versus* 0.04 e). More details on data collection and refinement are compiled in Tables 1 ▸ and 2 ▸.

## 3. Results and discussion   

### 3.1. Molecular structure   

**Figure 3:**
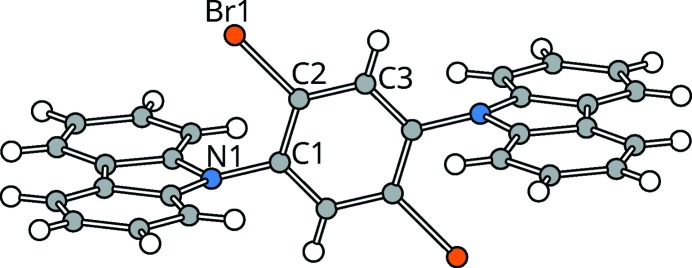
Geometry of molecule (1). C, N, Br and H atoms are represented by gray, blue, orange and white spheres of arbitrary radius. Coordinates taken from the 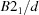 polytype.

Molecules of (1) (Fig. 3 ▸) in both polytypes possess 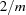 pseudo-symmetry (actual symmetry 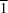 and *2*, respectively). The carbazole moieties are virtually planar [maximum distance to least-squares (LS) plane in the major 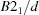 polytype: 0.045 (7) Å (N1)]. Remarkably, the C—N bond connecting the benzene and the carbazole is distinctly tilted with respect to the plane of the carbazole [angle of N(carbazole)—C(benzene) to the LS plane of carbazole: 15.5 (4)°].

### 3.2. OD description   

**Figure 4:**
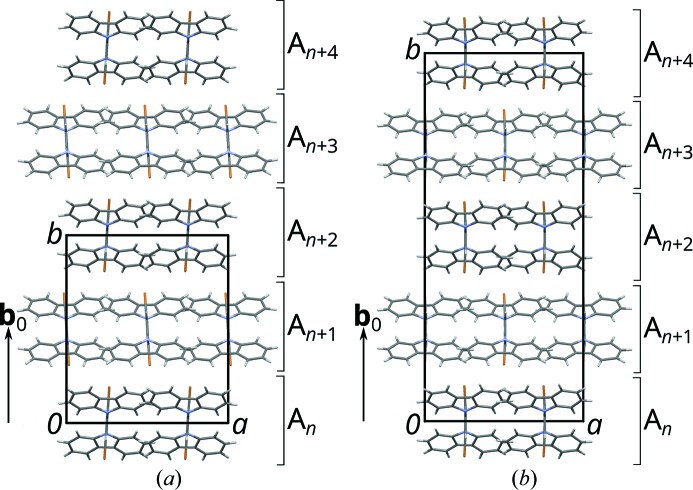
The (*a*) 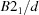 (MDO1) and (*b*) 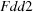 (MDO2) polytypes of (1) viewed down [001]. Color codes as in Fig. 3 ▸. Layer names are indicated to the right.

The crystal under investigation was made up of two polytypes (Fig. 4 ▸), each in two orientation states. The layers of the polytypes extend in the 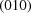 plane [here and in the following discussion all directions are given with respect to the *oB* setting 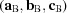 of §2.3], and will be designated as 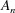, where *n* is a sequential number. The benzene rings are located at the center of these layers, the carbazoles at the interfaces.

An OD interpretation of polytypism is based on identifying pseudo-symmetry of these layers. The operations of these layers are called λ-partial operations (POs) (Dornberger-Schiff & Grell-Niemann, 1961 ▸). They are *partial* operations, because their domain of definition is a strict subset of Euclidean space 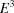. The operations relating different layers are likewise POs and are called σ-POs.

**Figure 5:**
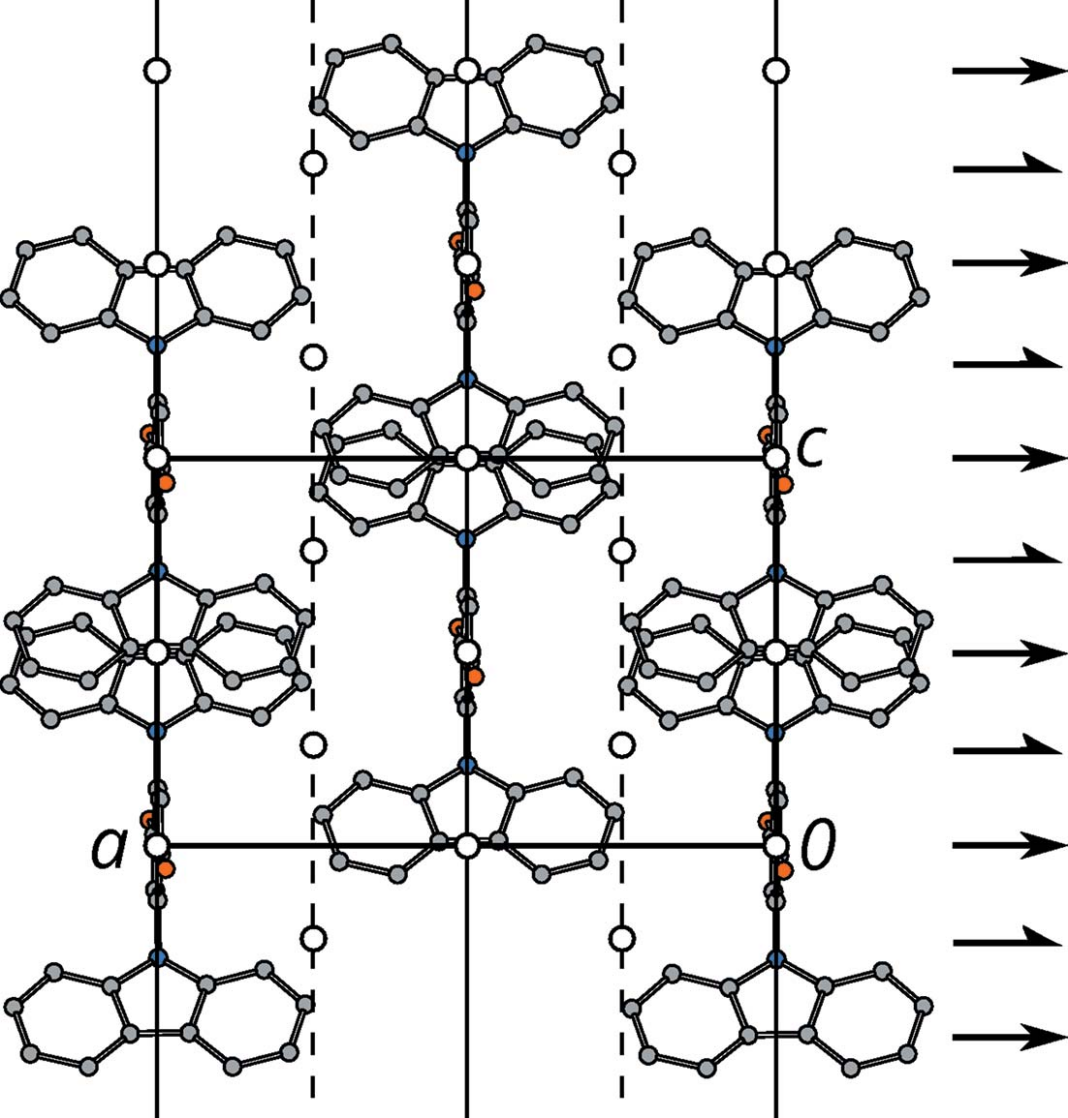
An *A_n_* layer viewed down the stacking direction [010]. Color codes as in Fig. 3 ▸. H atoms are omitted for clarity. (Pseudo)-symmetry operations are indicated using the usual graphical symbols (Hahn, 2006 ▸), the unit cell by a black rectangle. Coordinates taken from the 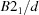 polytype.

Indeed, molecules (1) possess 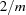 pseudo-symmetry with the twofold axis and the reflection plane in the 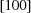 direction. By assuming perfect 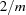 symmetry of the molecules, the 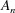 layers (Fig. 5 ▸) possess 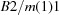 layer symmetry [in the tradition of OD literature parentheses are used to mark the direction lacking translational symmetry (Dornberger-Schiff & Grell-Niemann, 1961 ▸)]. Adjacent layers are related by a *d* glide reflection at a plane parallel to 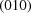.

Because the reflection planes of adjacent layers do not overlap, the structure belongs to an OD family of layers of one kind. Since the layers are non-polar with respect to the stacking direction, the OD family belongs to category I (Dornberger-Schiff & Grell-Niemann, 1961 ▸). The OD groupoid family symbol reads in the *oB* setting as
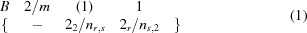
according to the notation of Dornberger-Schiff & Grell-Niemann (1961 ▸). The first line of the symbol indicates the 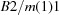 layer symmetry of the 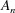 layers, the λ-POs. Below are listed the operations relating 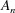 to 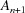 in *one possible* arrangement (σ-POs).


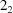
 is a generalization of the 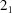 notation and describes a screw rotation with an intrinsic translation vector 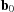, which is the vector perpendicular to the layer planes and the length of one layer width. In analogy, 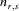 is a glide reflection with the intrinsic translation vector 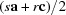 *etc*.

In the actual OD family of the title compound, the metric parameters describing the intrinsic translations of the σ-POs are 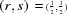. This can be written by the symbol
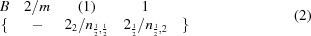
Here, the 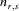 glide reflection becomes a 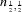 glide reflection, which is a *d* glide reflection with an intrinsic translation vector 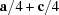, corresponding to a quarter of the face diagonal of the unit cell. Application of the *B* centering produces a second 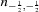 operation which is likewise a *d* glide reflection. On the other hand, no *d* glide reflections 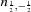 or 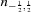 exist in this case.

The alternative stacking possibilities are determined by application of the NFZ relationship (Ďurovič, 1997 ▸). To choose the correct form of the NFZ relationship, it is crucial to realise that the 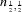 PO has *reverse continuations* (Dornberger-Schiff & Grell-Niemann, 1961 ▸), meaning that it maps 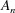 onto 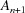, but also 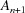 onto 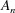. In other words, 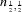 is a symmetry operations of the 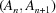 *pairs* of layers.

In such a case the NFZ relationship reads as 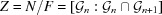, where 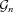 is the group of those operations of the 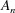 layer that do not reverse the orientation with respect to the stacking direction (λ-τ-POs in the OD literature). 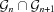 is the subgroup of those operations that are also valid for 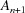. Thus, given a fixed 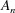 layer, the 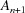 layer can appear in 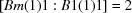 positions, which are related by the 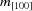 operation of the 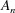 layer. In the alternative stacking possibility, the 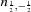 and 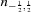 glide reflections are realised.

**Figure 6:**
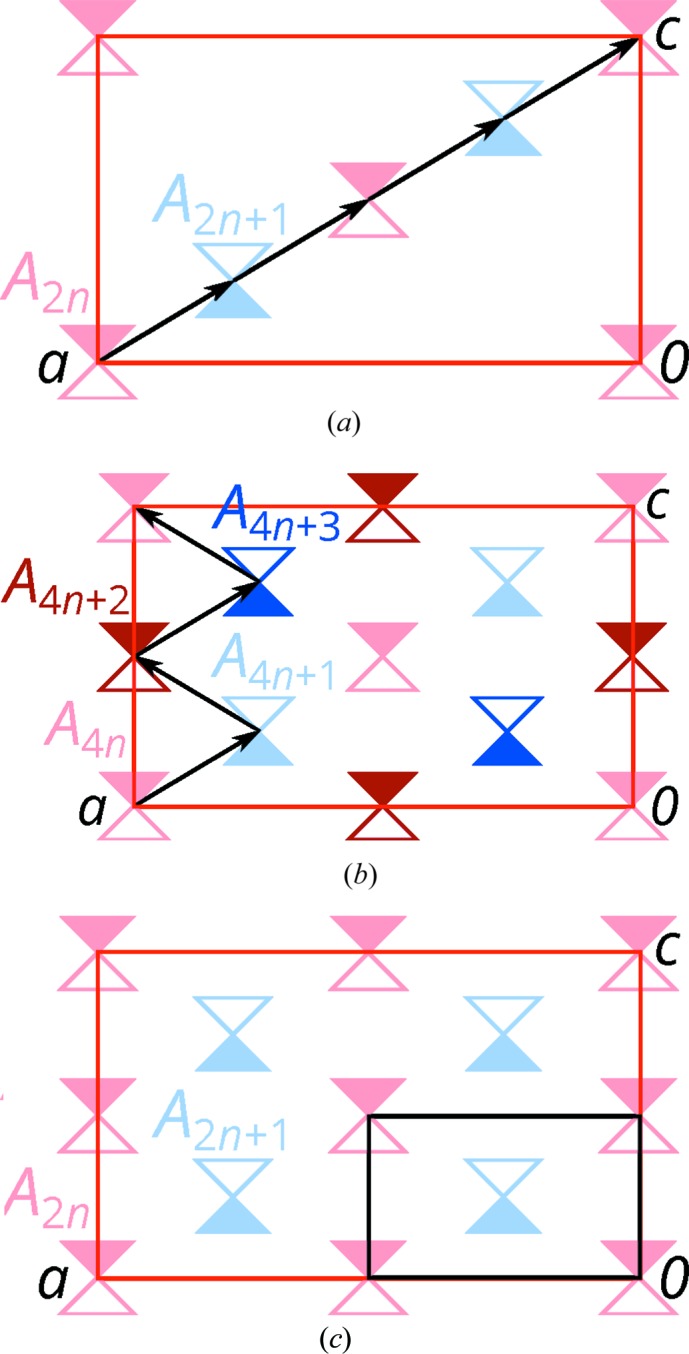
Schematic representation of the (*a*) MDO1 and (*b*) MDO2 polytypes, and (*c*) the family structure viewed down the stacking direction [010]. Molecules (1) with 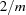 point symmetry are represented by triangles which are filled on one and outlined on the other side. Molecules in *A_n_* with even and odd *n* are red and blue, respectively. An additional translation component of 2**b**_0_ is indicated by darker colors. Black arrows indicate the intrinsic translation of the *d* glide reflection relating adjacent layers. The unit cells of the *A*_2*n*_ (*A*_4*n*_) layers are represented by a red rectangle, the unit cell of the family structure by a black rectangle.

By these stacking rules, the 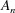 layers can be arranged to an infinity of different polytypes, which are all locally equivalent up to at least two layer widths. Of these, two polytypes are of a maximum degree of order (MDO), *i.e.* they cannot be decomposed into simpler polytypes (Dornberger-Schiff, 1982 ▸). MDO_1_ (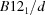, 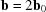, Fig. 6 ▸ *a*) is generated by repeated application of 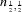 glide reflections; MDO_2_ (*F*2*dd*, 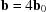, Fig. 6 ▸ *b*) by alternating application of 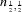 and 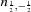 glide reflections. All other polytypes consist of fragments of the MDO_1_ and MDO_2_.

These two MDO polytypes make up the crystal under investigation. Indeed, experience shows that in the vast majority of observed cases, OD polytypes are of the MDO type. Identification of the MDO polytypes is therefore a crucial part of the interpretation of OD structures (Ďurovič, 1997 ▸).

### 3.3. Twinning and allotwinning   

To determine the possible orientation states that each polytype can adopt, the point group of the OD family (Fichtner, 1977 ▸), which is the point group generated by the linear parts of all POs of a member, is determined. In the title compound it is *mmm*. The point group of any member of the family is a subgroup of this point group.

The possible orientation states of a polytype are then determined by the coset decomposition of the point group of the member in the point group of the family. For the 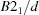 MDO_1_ polytype 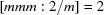 and there are therefore two orientation states. The coset decomposition is 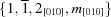 and 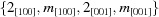. The latter is the twin law (Hahn & Klapper, 2006 ▸) relating the two orientations.

In analogy, for the *F*2*dd* MDO_2_ polytype 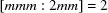 and there are again two orientation states described by the cosets 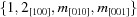 and 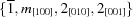. This twinning is by inversion and therefore determination of the twin volume ratio was only possible owing to the resonant scatterer Br.

### 3.4. Desymmetrization   

As is characteristic for ordered polytypes, the actual layers in both MDO polytypes are desymmetrized (Ďurovič, 1979 ▸) compared with the idealized OD description (the *prototype* layers). A too extreme desymmetrization casts doubt on an OD interpretation, because it means that the presumption of geometrically, and therefore energetically, equivalent layers is not valid.

In the MDO_1_ polytype, the symmetry of the 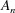 layers is related to the prototype layers by a *translationengleiche* symmetry reduction of index 2 from 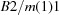 to 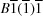. The point symmetry of the (1) molecules is reduced from 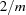 to 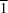. Of the σ-POs relating adjacent layers only the 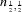 glide reflections and the 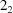 screw rotations are retained as symmetry operations of the whole polytype.

In the MDO_2_ polytype, the symmetry of the 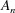 layers is related to the prototype layers by a *translationengleiche* symmetry reduction of index 2 from 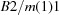 to 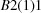. The symmetry of molecules (1) is 2. Of the σ-POs relating adjacent layers, the 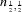 and the 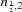 glide reflections are retained (the latter is likewise written as *d* in the *F*2*dd* symbol owing to the doubled *b*-axis).

**Figure 7:**
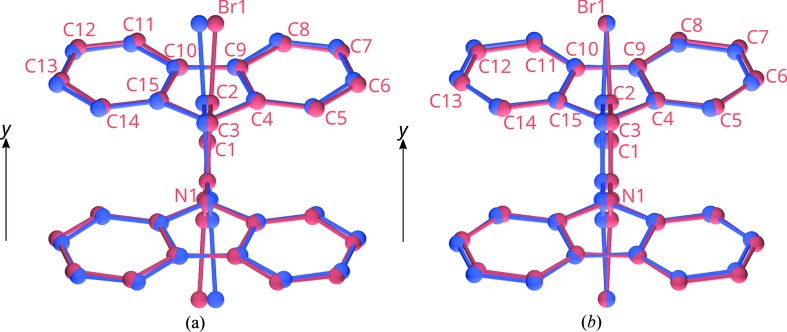
Excerpts of the overlap of an *A_n_* layer and its image by pseudo-symmetry in orthographic projection along [001] in (*a*) MDO1 and (*b*) MDO2. The atoms in both layers are painted in red and blue, respectively. Atom names are given for the molecule shown in red.

To quantify the desymmetrization, in both polytypes the lost symmetry was applied to an actual 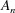 layer (in the MDO_1_ polytype the 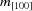 reflection; in the MDO_2_ polytype the inversion). An overlap of the actual 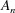 layers and their images are given in Fig. 7 ▸. The distance of the atoms mapped by pseudo-symmetry are compiled in Table 3 ▸.

The most striking difference in the desymmetrization of both polytypes pertains to the Br atom (MDO_1_: 0.435 Å, MDO_2_: 0.010 Å). In MDO_1_ the large deviation is due to a distinct inclination of the central dibromobenzene fragment of (1) with respect to the 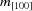 pseudo-reflection plane. In MDO_2_ on the other hand, the Br atom is located virtually on the pseudo-reflection plane (a *d* glide plane in the actual polytype) for chemical reasons. An inclination with respect to the reflection plane would result in a bent benzene ring, owing to the twofold rotation axis normal to the plane. The benzene rings are indeed apparently bent, though it has to be noted that the localization of the atoms in the MDO_2_ polytype is not exact owing to poor diffraction data (§3.5). The generally small deviations demonstrate the validity of the OD interpretation.

### 3.5. Diffraction pattern   

The family structure of an OD family is the fictitious structure in which all stacking possibilities of the family are realised (Ďurovič *et al.*, 2006 ▸). It plays a crucial role in the interpretation of the diffraction pattern of OD structures. The reflections of the family structure (*family reflections*) are always sharp (supposing that desymmetrization plays only a minor role). All polytypes contribute equally (proportional to the volume ratio) to the family reflections. The remaining reflections are only generated by certain polytypes and are therefore called *characteristic reflections*. They can be sharp if the polytypes are highly ordered, but may also be broadened in the case of frequent stacking faults.

The family structure of the OD family under investigation is derived by application of the 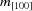 operation of the 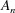 layers onto the 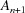 layers (Fig. 6 ▸ *c*). The symmetry of the family structure, the *superposition group* (Fichtner, 1977 ▸), is *Pmnn* with lattice basis 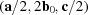. The symmetry of any stacking arrangement, ordered or disordered, is a subgroup of the superposition group.

In the reciprocal basis of the MDO_1_ polytype 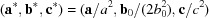 the family reflections are located at 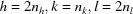 with 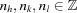. It can be shown (Ferraris *et al.*, 2008 ▸; Hans *et al.*, 2015 ▸) that if the layers are translationally equivalent, non-zero intensity on the rods with the family reflections is only observed for the family reflections, even for long-period polytypes and disordered stackings. Here, adjacent layers are not equivalent, but an analoguous result is obtained by decomposing the structure into 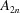 and 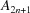 layers. Thus, characteristic reflections and diffuse scattering are only expected on rods 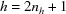, 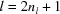. The characteristic reflections of both MDO polytypes are distinct. A summary of the reflection conditions is given in Table 4 ▸.

**Figure 8:**
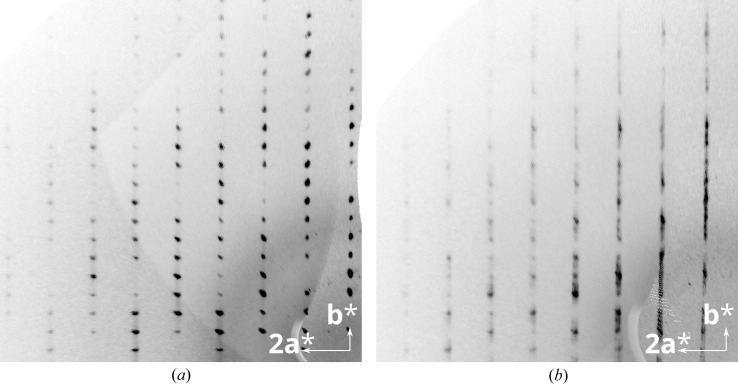
The (*a*) 2*kl* and (*b*) 3*kl* planes of reciprocal space of the crystal under investigation reconstructed from CCD data.

Indeed, in the actual diffraction patterns family reflections are sharp (Fig. 8 ▸ *a*), whereas the characteristic reflections (Fig. 8 ▸ *b*) are located on distinct streaks, indicative of disordered domains. The characteristic reflections are elongated in the stacking direction, *i.e.* the ordered domains are small. The intensities of the MDO_2_ characteristic reflections are weak and in the same order of magnitude as the intensities of the streaks.

A careful inspection of the family reflections reveals that they are likewise located on distinct, albeit very faint, streaks of diffuse scattering. This is due to the layers in the structure not being translationally equivalent as a consequence of desymmetrization (§3.4). Notably, the strongly diffracting Br atoms alternate between two positions in the MDO_1_ polytype and adopt a third orientation in the MDO_2_ polytype, where they are located close to the idealized positions. It can therefore be expected that in the disordered parts of the crystals the Br atoms feature a distinct modulation of their position, resulting in faint streaks.

### 3.6. Determination of the volume ratio   

The volume ratio of the four domains (MDO_1_:MDO_1_:MDO_2_:MDO_2_) was determined by a concurrent refinement (§2.3) as 36.6 (2):32.4 (2):14.9 (13):16.1 (12). Thus, the MDO_1_:MDO_2_ ratio is 69.0:31.0. As expected for a crystal with a high stacking fault probability, the volume fractions of the two orientations of each polytype are approximately equal. The ESU of the fractions of the MDO_2_ domains is significantly higher, because it is derived from the anomalous scattering of the Br atoms.

The refinement was performed under the assumption of ideal (*allo*)twinning. In such a case the individual domains diffract independently and the intensities are calculated as the weighted sums of the intensities of the individuals. Given the signs of disorder and small *ordered* domains (§3.5), this assumption is only valid to a certain degree. Moreover, the intensities of the MDO_2_ characteristic reflections are in the order of magnitude of the intensities of the streaks and therefore the determined volume ratio has to be considered as inexact.

For comparison, the polytype fractions were also indirectly determined by a refinement using only the MDO_1_ reflections. The twin volume fraction, which describes the ratio of both orientations of the MDO_1_ polytype, refined to 53.1:46.9 (2). Indeed, owing to the laws of probability, volume fractions in a polysynthetic twin with a high stacking fault probability are expected to be close to equal. The Br position was refined as occupationally disordered (§2.3). As we have shown for KAgCO_3_ (Hans *et al.*, 2015 ▸), the actual fraction of the major polytype is 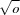, where *o* is the occupancy of the major Br position, if two conditions hold: Firstly, the domains diffract independently and secondly the contributions of both polytypes to the family reflections are equal. As noted above, we cannot ascertain the former and also the latter is certainly not perfectly fulfilled owing to the strong desymmetrization of the Br position. Nevertheless, the refined occupancy ratio of the Br atoms is 92.25:7.75 (14), which corresponds to an MDO_1_:MDO_2_ ratio of 72.16:27.84. The similarity of the ratios derived from both refinements indicates that the assumption of independent diffraction is mostly valid.

### 3.7. Comparison to KAgCO_3_   

In two instances the (1) allotwins differ notably from the KAgCO_3_ allotwins that we have described previously (Hans *et al.*, 2015 ▸). Firstly, KAgCO_3_ featured a high degree of order and large domains, as witnessed by sharp reflections and an absence of diffuse scattering. Secondly, KAgCO_3_ featured virtually no desymmetrization and therefore contributions to the family reflections can be assumed to be equal for all polytypes. These two points are seemingly paradoxical, because one would expect that distinct polytypes are stabilized by desymmetrization. Hence, it is shown that crystallization is a complex dynamic process, which cannot be broken down to such simple rules.

## 4. Conclusion and outlook   

The crystals of (1) are a further addition to the growing set of known allotwins and here it should be stressed again that the phenomenon is more common than one might expect. It is therefore crucial to recognize and pursue the, often subtle, signs of allotwinning like peaks in the difference Fourier maps and weak additional reflections. Moreover, we want to emphasize the diversity of the phenomenon, making it necessary to address every allotwin as a unique problem.

In a final, and longer, remark it is noted that the OD structure of (1) is particularly unusual owing to the metric parameters 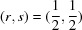. Only for this particular pair of parameters the MDO polytypes possess 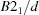 and *F*2*dd* symmetry.

OD groupoid families, which are the only accepted symmetry-classification system of OD families, disregard these metric parameters, despite their fundamental importance. Indeed, to the OD groupoid family of (1) [equation (1)] also belong the groupoids of fully ordered OD structures (*i.e.* with only one structure in the family). It therefore seems necessary to develop a finer classification system.

**Figure 9:**
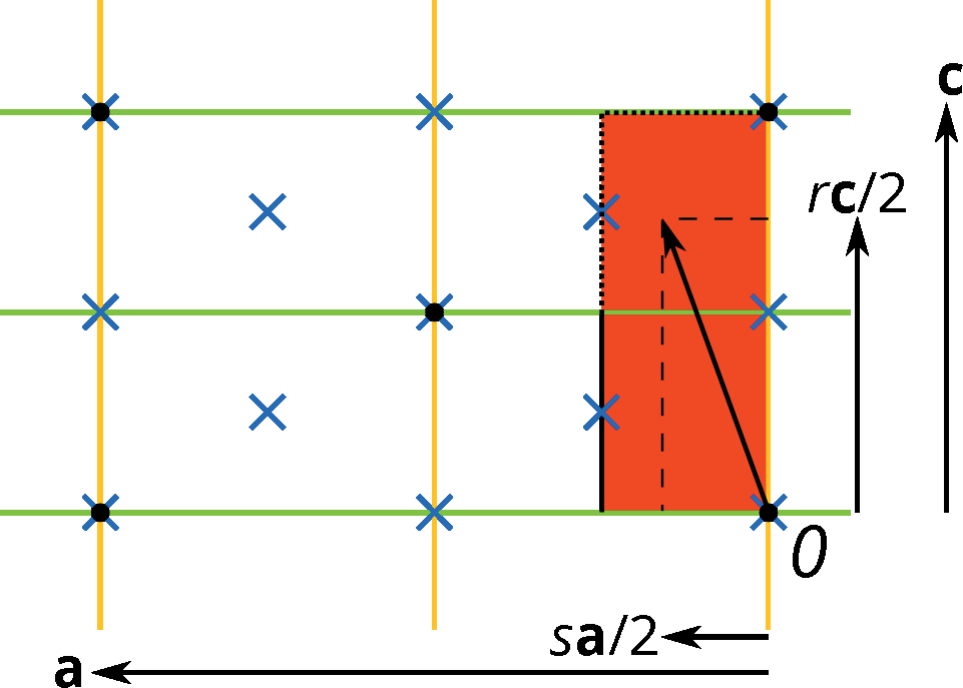
Special values of the intrinsic translation vector of the 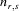 σ-PO. 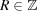: green lines; 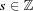: yellow lines; 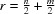, 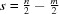 with 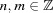: blue crosses. Lattice nodes are represented by black dots. The set of translation vectors to be considered are represented by a red rectangle. At the dotted lines the set opens, at the continuous lines it is closed. An intrinsic translation vector 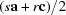 and its components 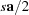 and 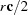 are indicated.

On the example of the OD groupoid family of (1), a short overview of the special values that the metric parameters can adopt will be developed. First, one has to realise that for a given 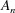 layer different 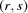 pairs describe the same OD family. Notably, the intrinsic translation of the 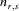 glide reflection need only be considered modulo the rectangular centered (*ob*) lattice of the layers. Moreover, owing to the group of λ-τ-POs [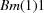], if 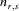 is a σ-PO in an alternative stacking arrangement 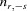 is also a σ-PO. Therefore, for a given 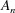 layer, the pairs 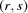 and 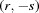 describe the same family of structures. In total, for this OD groupoid family the intrinsic translation vectors of the 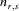 glide reflection can be limited to the asymmetric unit of the 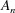 layers. The corresponding metric parameters are 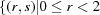, 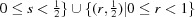 (Fig. 9 ▸). Note that in other OD families conjugation of σ-POs with λ-τ-POs may alter the linear part, leading to more complex situations.

As has been discussed above (§3.2), two factors determine the number *Z* of stacking possibilities. Firstly, whether the 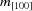 glide planes of adjacent layers overlap. This is generally the case for 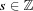. With the restrictions on 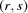 above, the cases 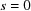, 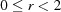 remain (Fig. 9 ▸). The second criterion is whether there exists a reverse continuation. The 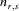 σ-PO in the 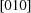 direction is a reverse continuation if 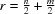, 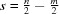, 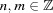 (*i.e.* *r* and *s* are integral multiples of 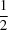 and 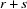 is an integer). With the restrictions on 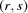 above there are three 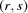 pairs to consider, *viz.* 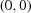, 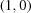, 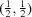. The 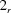 σ-PO is a reverse continuation if 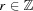. With the restrictions above, these cases are 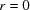, 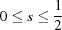 and 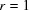, 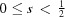. These three sets and their intersections are summarized in Table 5 ▸. There are three values that *Z* can adopt: 1 (the structure is fully ordered), 2 and 4. The fundamental importance of these sets of parameters is demonstrated by the symmetry of the MDO polytypes (rightmost column in Table 5 ▸).

More complexities arise when considering the symmetry of the family structure. Here, one has to differentiate between rational and irrational metric parameters. If irrational, the lattice of the family structure may become dense in one or two directions.

Certainly, devising a proper definition of the different classes of metric parameters and their tabulation will be a difficult task. Nevertheless, given the frequent occurrence of OD structures, it is overdue.

## Supplementary Material

Crystal structure: contains datablock(s) mon, orth. DOI: 10.1107/S2052520616018291/wf5130sup1.cif

Structure factors: contains datablock(s) mon. DOI: 10.1107/S2052520616018291/wf5130monsup2.hkl

Structure factors: contains datablock(s) orth. DOI: 10.1107/S2052520616018291/wf5130orthsup3.hkl

CCDC references: 1517283, 1517284

## Figures and Tables

**Table 1:** Experimental details

Crystal data	
*T* (K)	100
θ range (°)	2.41–27.88
Crystal description, color	Plate, yellow
Crystal size (mm)	0.02 × 0.24 × 0.36
	
Data collection	
Diffractometer	Bruker KAPPA APEX II CCD
Absorption correction	Multi-scan, *SADABS*
*T*_min_, *T*_max_	0.36, 0.93
No. of measured, independent and observed reflections [*I* ≥ 3σ(*I*)] reflections	7563, 4385, 3676
*R*_int_ (point group 2)	0.0423
	
Refinement	
*R*[*F* ^2^ > 2σ(*F* ^2^)], *wR*(*F*), *S*	0.055, 0.072, 1.81
No. of parameters, restraints	231, 0

**Table 2:** Structural data of both polytypes of (1)

	Monoclinic polytype	Orthorhombic polytype
Crystal data		
Chemical formula	C_30_H_18_Br_2_N_2_	C_30_H_18_Br_2_N_2_
*M*_r_	566.3	566.3
Crystal system, space group	Monoclinic, 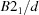	Orthorhombic, 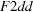
*a*, *b*, *c* (Å)	14.5394 (13), 16.8717 (14), 9.0877 (8)	14.5394 (13), 33.743 (3), 9.0877 (8)
β (°)	90	90
*V* (Å^3^)	2229.3 (3)	4458.5 (7)
*Z*, *Z*′	4, 0.5	8, 0.5
Range of *h*, *k*, *l*	*h* = −19 → 19, *k* = −22 → 18, *l* = −9 → 11	*h* = −19 → 19, *k* = −44 → 37, *l* = −9 → 11
		
Refinement		
Δρ_max_, Δρ_min_ (e Å^−3^)†	1.47, −0.63	1.76, −1.29
Twin operation	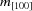	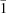
Twin volume fractions‡	0.366 (2), 0.3239 (19)	0.149 (13), 0.161 (12)

† *F*_obs_ attributed to domains according to *F*_calc_ ratios.

‡ Fractions of the whole edifice.

**Table 3:** Distance, *d*, of atoms in an actual *A_n_* layer to the closest atoms in the image of the same layer by idealized symmetry (MDO_1_: *m*_[100]_, MDO_2_: 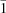)

		*d* (Å)	
Atom	Atom	MDO_1_	MDO_2_
Br1	Br1	0.435	0.010
N1	N1	0.061	0.140
C1	C1	0.015	0.174
C2	C2	0.189	0.166
C3	C3	0.131	0.204
C4	C15	0.105	0.060
C5	C14	0.116	0.139
C6	C13	0.176	0.149
C7	C12	0.191	0.150
C8	C11	0.185	0.093
C9	C10	0.128	0.053

**Table 4:** Integral reflection conditions of both MDO polytypes and the family structure with respect to the reciprocal base (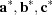) = 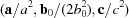; 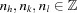

Polytype	Symmetry, lattice basis	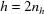 , 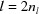	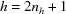 , 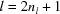
MDO_1_	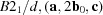	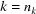	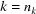
MDO_2_	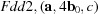	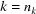	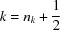
Family structure	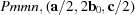	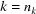	–

**Table 5:** (*r*, *s*) pairs of metric parameters of the OD groupoid family in equation (1) featuring different kinds of stacking arrangements and MDO polytypes

Parameters	*Z*	Reverse continuous scan	*m*_[010]_ overlap	MDO polytypes
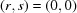	*N*/*F* = 1	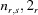	Yes	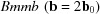
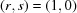	*N*/*F* = 1	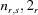	Yes	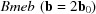
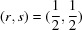	*N*/*F* = 2	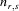	No	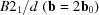 , 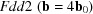
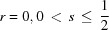 ; 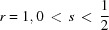	*N*/*F* = 2	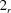	No	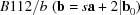 , 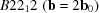
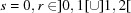	2*N*/*F* = 2	–	Yes	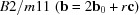 , 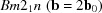
Other	2*N*/*F* = 4	–	No	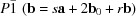 , 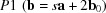 , 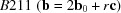 , 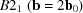

## References

[bb1] Bruker (2014). *APEXII, RLATT, SAINT, SADABS* and *TWINABS.* Bruker AXS Inc., Madison, Wisconsin, USA.

[bb2] Burzlaff, H. & Zimmermann, H. (2006). *International Tables for Crystallography*, Vol. A, Ch. 9.1, pp. 742–749. Chester: IUCr.

[bb3] Dornberger-Schiff, K. (1982). *Acta Cryst.* A**38**, 483–491.

[bb4] Dornberger-Schiff, K. & Grell-Niemann, H. (1961). *Acta Cryst.* **14**, 167–177.

[bb5] Ďurovič, S. (1979). *Krist. Techn.* **14**, 1047–1053.

[bb6] Ďurovič, S. (1997). *EMU Notes Mineral.* **1**, 3–28.

[bb7] Ďurovič, S., Krishna, P. & Pandey, D. (2006). *International Tables for Crystallography*, Vol. C, Ch 9.2, pp. 752–773. Chester: IUCr.

[bb8] Ferraris, G., Makovicky, E. & Merlino, S. (2008). *IUCr Monographs on Crystallography*, Vol. 15. Oxford University Press.

[bb9] Fichtner, K. (1977). *Beitr. z. Algebra u. Geometrie*, **6**, 71–99.

[bb10] Friedel, G. (1904). *Bulletin de la Société d’Industrie Minérale*, Vol. Quatrième Serie, Chap. Tomes III et IV, pp. 393–448. Saint Etienne: Imprimerie Théolier J. et Cie.

[bb11] Friese, K., Hönnerscheid, A. & Jansen, M. (2003). *Z. Kristallogr.* **218**, 536–541.

[bb12] Grell, H. (1984). *Acta Cryst.* A**40**, 95–99.

[bb13] Hahn, T. (2006). *International Tables For Crystallography*, Vol. A, Ch. 1.4, pp. 7–11. Chester: IUCr.

[bb14] Hahn, T. & Klapper, H. (2006). *International Tables For Crystallography* Vol. D, Ch. 3.3, pp. 393–448. Chester: IUCr.

[bb15] Hans, P., Stöger, B., Weil, M. & Zobetz, E. (2015). *Acta Cryst.* B**71**, 194–202. 10.1107/S2052520615004138 25827372

[bb16] Jahangiri, A., Fleckhaus, A., Lidin, S. & Strand, D. (2013). *Acta Cryst.* B**69**, 509–513. 10.1107/S2052519213018605 24056360

[bb22] Krüger, H., Kahlenberg, V., Petříček, V., Phillipp, F. & Wertl, W. (2009). *J. Solid State Chem.* **182**, 1515–1523.

[bb17] Nespolo, M. & Ferraris, G. (2001). *Eur. J. Miner.* **13**, 1035–1045.

[bb18] Nespolo, M., Kogure, T. & Ferraris, G. (1999). *Z. Kristallogr.* **214**, 5–8.

[bb23] Petříček, V., Dušek, M. & Palatinus, L. (2014). *Z. Kristallogr.* **229**, 345–352.

[bb19] Sheldrick, G. M. (2015). *Acta Cryst.* A**71**, 3–8.

[bb20] Spek, A. L. (2009). *Acta Cryst.* D**65**, 148–155. 10.1107/S090744490804362X PMC2631630 19171970

[bb21] Stöger, B., Holzhacker, H. & Kirchner, K. (2015). *Z. Kristallogr.* **230**, 621–628.

